# Abnormal functional hubs in migraine patients: a resting-state MRI analysis about voxel-wise degree centrality

**DOI:** 10.22514/jofph.2024.006

**Published:** 2024-03-12

**Authors:** Yi-Chao Chen, Zi-Min Cao, Guo-Yun Liu, Zhi-Jun Li, An-Qi Shi, Jing-Nan Jia, Jian-Wei Huo, Jun Wang, Chao-Qun Yan

**Affiliations:** ^1^Department of Acupuncture and Moxibustion, Dongzhimen Hospital Beijing University of Chinese Medicine, 100700 Beijing, China; ^2^Department of Radiology, Beijing Hospital of Traditional Chinese Medicine affiliated to Capital Medical University, 100010 Beijing, China

**Keywords:** Migraine, Pain, Degree centrality, Functional connectivity, Resting-state fMRI

## Abstract

The purpose was to explore the spatial centrality of the whole brain functional network related to migraine and to investigate the potential functional hubs associated with migraine. 32 migraine patients and 55 healthy controls were recruited and they received resting-state functional magnetic resonance imaging voluntarily. Voxel-wise Degree Centrality (DC) was measured across the whole brain, and group differences in DC were compared. False Discovery Rate and permutation test (5000 times) were used for multiple comparisons. Finally, significant differences in functional connectivity (FC) between seeds and other brain regions were further researched by the seed-based approach. The correlation analyses between the changes in the brain function and clinical features were also performed. The results showed that, compared to healthy controls, migraine patients exhibited significantly increased DC in the left anterior cingulate cortex (ACC), slightly increased DC in the right ACC and the right medial superior frontal gyrus (SFG). No significant correlation was found between DC and clinical variables. The seed-based analyses showed that migraine patients showed increased FC between the right SFG and left ACC, decreased FC between the left ACC and left superior temporal gyrus (STG). FC value of the right SFG was positively correlated with the score of migraine-specific quality-of-life questionnaire about role in function-preventive in migraine patients. According to relatively changed DC, we found that migraine patients exhibited specific abnormal intrinsic functional hubs. These findings expand our understanding of functional characteristics of migraine, and may provide new insights into understanding the dysfunction and pathophysiology of migraine patients.

## 1. Introduction

Migraine has been understood as a common neurological disorder, featured with recurrent moderate to severe headache accompanied with gastrointestinal, neurological and sensory functional symptoms and often recrudesces in daily life [1]. Furthermore, migraine can give rise to more comorbid psychiatric diseases, such as depression and anxiety [2]. According to global burden of headache reports in 2018, approximately 1.04 billion individuals suffered from a migraine attack, with global age-standardized prevalence 14.4% overall (18.9% were female and 9.8% were male). Because migraine had a much higher disability weight, it caused approximately 45.1 million individuals living with disability globally [3]. Until now, migraine has been regarded as the leading cause of disability under the age of 50 years [4]. Migraine not only causes severe physical and mental suffering but also has a significant damage to the social economy. For the European Union, headache disorders are primary health-related drivers of tremendous economic losses, which has immediate adverse impact for healthcare policy [5]. In the United States of America, patients and their employers have to shoulder a heavy financial burden of migraine in the manifestation of lost productivity and bedridden days [6].

Rest-state functional magnetic resonance imaging (rs-fMRI) is an imaging technique that relies on changes in blood oxygen levels to obtain a functional map of brain activity in the resting state of the subjects (maintaining stillness and not performing any structured cognitive tasks) [7, 8, 9]. The imaging technology has the characteristics of safety, non-invasive, high repeatability, less interference and high spatial resolution [10, 11, 12]. Nowadays, a number of studies have used the functional magnetic resonance imaging (fMRI) to delve deeper into brain changes on migraine. For example, Lingling Dai *et al*. [13] by using rs-fMRI and voxel-wise FC density analysis and examining the large-scale FC pattern over the whole brain in 17 patients with chronic migraine without medication overuse and 35 healthy controls, observed that local FC density in the right dorsal ACC was positively correlated with headache intensity. Yingxia Zhang *et al*. [14] employed whole brain functional connectivity homogeneity (FcHo) method to identify the voxel-wise changes of FC patterns in 21 patients with migraine without aura and 21 gender and age matched healthy controls and found the decreased FcHo values in the left anterior insula and decreased FC between the left anterior insula and anterior cingulate cortex. Many previous neurological studies about migraine has confirmed that the trigeminovascular nociceptive pathway with a disturbed homeostasis is a crucial factor for susceptibility to migraine and the pathophysiology between vascular control and the brainstem nuclei regulating antinociception, especially in the ventrolateral periaqueductal gray (PAG) could show a state of imbalance in activity [15, 16, 17]. In addition, several animal studies also indicate descending modulation of the trigeminocervical complex (TCC), mainly through the ventrolateral PAG and rostral ventromedial medulla (RVM), could cause both the activation of “on” cells and the inhibition of “off” cells in the RVM, and this kind of change is critical for activation of TCC and aggravation of migraine headache [18, 19]. However, the exact neurological basis of pathomechanism in patients with migraine is largely lacking in sufficient research evidence to support for other functional hubs. Therefore, there is no consensus in abnormal functional hubs in migraine patients.

Fortunately, neuroimaging methodologies can be helpful to explore the changes in the whole brain, and to refine our understanding of pathomechanism in the patients with migraine. In this study, our aim was to describe the spatial centrality distribution (hubs) of the whole brain functional network and to identify the potential altered inherent functional hubs in patients with migraine by using voxel-wise degree centrality (DC) and seed-based functional connectivity (FC) analysis. We hypothesized that patients with migraine may show the abnormal strengthened or weakened connectivity of the inherent functional hubs across the whole brain.

## 2. Materials and methods

### 2.1 Participants

From August 2020 to May 2021, migraine patients were recruited from outpatient clinics in the Department of Acupuncture and Moxibustion of Dongzhimen Hospital, Beijing University of Chinese Medicine, by displaying recruitment posters outside the clinics.

The inclusion criteria for migraine patients were as follows: (i) according to the International Classification of Headache Disorders, 3rd edition, meeting the International Headache Society diagnostic criteria 1.1 or 1.2.1 [1, 20]; (ii) male or female aged from 18 to 65 years; (iii) initial onset of migraine before 50 years of age; (iv) a history of disabling migraine for at least 1 year (migraine attack occurred ≥2 times per month and the duration of each migraine attack ≥4 h); (v) in the past 3 months, the average number of migraine attacks every 4 weeks is 2–8 times (including 2 and 8 times); (vi) not taking any drugs that have the effect of preventing migraine in the past month, such as β-blockers, calcium channel blockers, antiepileptic drugs, antidepressants and 5-hydroxytryptamine (5-HT) receptor blockers, *etc.*; (vii) ability to complete the headache diary and sign the informed consent as required.

The criteria for exclusion were as follows: (i) headache caused by organic diseases (such as subarachnoid hemorrhage, cerebral hemorrhage, cerebral embolism, cerebral thrombosis, vascular malformations, arteritis, hypertension or arteriosclerosis, *etc.*); (ii) the presence of neurological diseases, immune deficiency, bleeding disorders or allergies; (iii) using drugs to control migraine attacks within 1 month before enrollment; (iv) alcohol allergy, alcohol or other drug abusers; (v) participants in other clinical trials at the same time; (vi) pregnancy or lactation women, or those who plan to become pregnant within 6 months; (vii) any contraindications to MRI scans (such as pacemakers, aneurysm clips, artificial heart valves, ear implants or metal fragments, foreign bodies in the eyes, skin or body).

Healthy controls were matched to migraine patients for age, sex and education-level, and were recruited from the community through advertising. Inclusion criteria for healthy controls were as follows: (i) male or female age from 18 to 65 years; (ii) no experience of regular migraine attacks before, and not meeting the International Headache Society diagnostic criteria 1.1 or 1.2.1; (iii) absence of significant heart disease, lung disease, neurological or major psychiatric disorders; (iv) no magnetic resonance imaging examination contraindications such as pacemakers, defibrillators, vascular clips, implantable electrical or magnetic devices, mechanical heart valves, cochlear implants, *etc.* in the body and those who are not claustrophobic.

In this study, migraine patients and healthy controls were diagnosed by two specialized physicians who were trained in migraine prior to the study. Brain imaging data were collected from all migraine patients and healthy controls. Before fMRI scanning, all participants completed a packet of questionnaires including demographic data, Self-rating Anxiety Scale (SAS), the Self-rating Depression Scale (SDS) and Edinburgh Handedness Inventory [21]. In addition to the above questionnaires, migraine patients also need to fill in Migraine-Specific Quality-of-Life Questionnaire (MSQ) [22, 23, 24], the average Visual Analogue Scale (VAS) [25] and frequencies of migraine attacks and taking analgesic based on the pain in the past four weeks before scanning.

### 2.2 MRI data acquisition

All magnetic resonance imaging (MRI) scanning was conducted on a Siemens 3.0 Tesla scanner (Skyra, Siemens, Erlangen, Germany) in the Department of Radiology for Beijing Hospital of Traditional Chinese Medicine Affiliated to Capital Medical University for acquiring better MRI Data. Before MRI scanning, participants were asked to check any carryon magnetic metal items and electronic equipment to keep safety and then remain supine with their head tightly and comfortably fixed by foam pads and straps to reduce head movement as much as possible. In order to reduce brain activity [26], participants were required to stay still and keep an eye on a cross-shaped icon on the screen during fMRI scanning. And during T1-weighted and T2-weighted imaging scanning, participants were not asked to do so. Every scanning session lasted approximately 30 mins. A high-resolution T1-weighted magnetization prepared rapid gradient echo (MPRAGE) sequence was acquired and covered the entire brain sagittal slices, repetition time (TR) = 2300 ms, echo time (TE) = 2.32 ms, field of view (FOV) = 240 × 240 mm, flip angle = 8°, acquisition matrix = 256 × 256, 290 scans. Rs-fMRI data were acquired by using a single shot gradient echo-planar imaging (EPI) sequence (40 axial slices, TR = 3000 ms, TE = 30 ms, FOV = 220 × 220 mm, flip angle = 90°).

### 2.3 Imaging data preprocessing

All the conventional T1-weighted and T2-weighted imaging were reviewed prior to preprocessing by two senior radiologists in the department of radiology of Beijing Hospital of Traditional Chinese Medicine Affiliated to Capital Medical University in order to exclude macrostructural brain lesions that might affect brain function or microstructure. The two senior radiologists had displayed and checked all the high-resolution T1-weighted images carefully, and checked functional images by using MRIcro software (www.MRIcro.com) to rule out potential low-quality images. None of the participants were excluded due to brain lesions or poor image quality.

The resting-state functional images were preprocessed by using Data Processing & Analysis for Brain Imaging (DPABI) (http://rfmri.org/DPABI) and Statistical Parametric Mapping (SPM8) (http://www.fil.ion.ucl.ac.uk/spm). The first 10 functional volumes of each scanning were needed to remove in order to adapt the participants to the noise of scanning and ensure the stability of initial signal and the completion of slice timing. Three-dimensional head motion correction was performed for the remaining time points. No participants were ruled out based on the head motion criteria, which included the maximum head rotation of less than 2.0° on any axis and the maximum head movement of less than 2.0 mm on any axis [27]. The Diffeomorphic Anatomical Registration Through Exponentiated Lie Algebra tool was applied to compute transformations from native space to Montreal Neurological Institute (MNI) space, and resampled to 3 mm × 3 mm × 3 mm voxels. Subsequently, the white matter signal, cerebrospinal fluid signal, global signal, and Friston 24-parameter were regressed from the time series of all voxels *via* linear regression [28]. Finally, a temporal filter (0.01–0.08 Hz) was performed to reduce the effect of low-frequency drift and high-frequency noise [29].

### 2.4 Voxel-wise degree centrality analysis

Resting-State fMRI Data Analysis Toolkit DPABI was used to calculate the voxel-wise DC for the resting-state fMRI time series in the predefined gray matter mask provided by DPABI in the MNI-152 standard space, 67541 voxels. Each voxel acted as a node, and all pairs of voxel correlations as the edge. For each participant, the Pearson’s correlation coefficients between any pairs of voxels within the predefined mask were calculate to accordingly construct functional connectivity matrix of the whole brain. Based on the adjacency matrix of a graph, voxel-wise DC can be calculated as in Eqn. (1) [30].

$$\text{DC}\left(i\right)=\sum_{j=1}^{N}r_{\text{ij}}\left(r_{\text{ij}}>r_{0}\right)$$

Where the correlation coefficient between voxel i and voxel j is expressed as r_ij_ and the term r_0_ acts as a correlation threshold and its main function is estimating the weak correlation [30, 31, 32]. Different correlation thresholds (r_0_ = 0.15, 0.2, 0.25 and 0.3) were calculated in this study [33, 34]. In order to improve normality, the resulting individual DC maps were converted into Fisher z score maps, which specifically means that individual correlation matrices were transformed into a z score matrix using Fisher’s r-to-z transformation. The normalized functional images (z score maps) were smoothed spatially with a 6 mm full-width-at-half-maximum Gaussian kernel.

### 2.5 Seed-based functional connectivity analysis

In order to further explore variations about resting-state FC, we performed the seed-based FC analysis. The average time series was obtained from the seed and the correlation analysis was conducted in a voxel-wise way using Data Processing Assistant for Resting-State fMRI software package (DPARSF, advanced version, http://www.rfmri.org) [35]. Regions with altered DC value of the left anterior cingulate cortex (ACC) between migraine patients and healthy controls were defined as seeding areas in the FC analysis. A correlation map of seed was obtained by correlation analysis between the reference time series and the time series of the rest of the brain in a voxel-wise manner. Finally, the resultant correlation maps were transformed to z-score maps using Fisher’s r-to-z transformation.

### 2.6 Statistical analysis

The demographic and clinical data differences between the migraine patients and healthy controls were computed using the IBM Statistical Package for the Social Sciences 25.0 software (IBM SPSS Inc., Chicago, IL, USA). We set the threshold for statistical significance at *p *< 0.05 and all hypothesis tests were two-tailed. The Shapiro-Wilk test was applied to tests of data normality, and observations of histograms were made. Independent two-sample *t*-tests were applied to analyze continuous variables with normal distribution. Otherwise, a Mann-Whitney U statistic was applied to analyze the data with non-normal distribution. For categorical variables, the chi-square (χ^2^) test was applied to analyze the data of gender ratios.

For the voxel-wise DC and seed-based FC, we performed multiple regression to process the data, regressed from covariables such as age, gender and education. After that, independent two-sample *t*-tests were performed to assess differences of regressed results between groups in the voxel-wise DC. For multiple comparisons, the permutation test (5000 times) with False Discovery Rate (FDR) [36, 37, 38] corrected *p *< 0.05 was considered statistically significant. The association between values of DC and clinical variables was examined by Pearson’s correlation analysis with a statistical significance level of *p *< 0.05.

## 3. Results

We totally recruited 87 participants from August 2020 to May 2021. All the participants fulfilled the inclusion and no migraine patients or healthy controls were excluded or voluntarily dropped out. Among these participants, migraine patients had a history of migraine for more than 1 year (migraine attack occurred ≥2 times per month and the duration of each migraine attack ≥4 h) and healthy controls had no history of migraine, tension headache and other types of headaches. During the study, no participants experienced safety events such as chest tightness, palpitations, nausea, injuries, burns or even deaths. All demographic, clinical and imaging data of all participants were well preserved and not lost. Therefore, data from 87 participants were included in the analysis.

### 3.1 Demographic and clinical data

Demographic and clinical data of two groups are presented in Table 1. In our study, 55 participants are healthy controls (male 20, female 35) and 32 participants are migraine patients (male 7, female 25). According to the analysis of Edinburgh Handedness Inventory [21], all participants are right-handed. In Table 1, analysis of demographic variables revealed that there were no significant differences between the migraine patients and healthy controls in age (*p* = 0.420 > 0.05, independent two-sample *t*-tests), years of education (*p* = 0.105 > 0.05, independent two-sample *t*-tests) and gender (*p* = 0.159 > 0.05, Pearson chi-square tests) and significant differences were observed in SAS and SDS between the migraine patients and healthy controls (all *p *< 0.05, independent two-sample *t*-tests). Migraine patients’ data of MSQ, VAS, frequencies of migraine attacks and taking analgesic are presented in Table 2.

**Table 1.** Demographic and clinical data between healthy controls and migraine patients.

Characteristic	Healthy controls (N = 55)		Migraine patients (N = 32)		95% CI	*p*-value*
	Mean (min to max)	SD	Mean (min to max)	SD		
Age (year)	39.62 (23 to 71)	16.309	37.03 (19 to 64)	13.785	−4.237 to 9.411	0.453
Education (year)	15.51 (7 to 23)	3.731	16.66 (9 to 20)	2.755	−2.541 to 0.246	0.105
SAS	33.87 (25 to 48)	7.290	44.16 (27 to 58)	7.467	−13.535 to −7.032	<0.001
SDS	34.85 (25 to 53)	8.191	43.41 (27 to 60)	9.112	−12.326 to −4.777	<0.001

Abbreviations: N: number; 95% CI: 95% Confidence Interval; SD: standard deviation; SAS: Self-Rating Anxiety Scale; SDS: Self-Rating Depression Scale. All the above data satisfy normal distribution. *The Shapiro-Wilk test was used to test the assumption of normal distribution and all data are normally distributed.

**Table 2.** Characteristic information of migraine patients.

Characteristic	Mean (min to max)	SD
Frequencies of migraine attacks (each 4 weeks)	3.97 (2 to 8)	1.892
Frequencies of taking analgesics (each 4 weeks)	2.75 (0 to 8)	2.410
VAS (average of the last four weeks)	6.94 (5 to 9)	1.045
MSQ-E	75.83 (40 to 100)	15.681
MSQ-P	71.16 (25 to 100)	15.373
MSQ-R	60.36 (40 to 80)	10.385

*Abbreviations: VAS: visual analogue scale; MSQ-E: Migraine-Specific Quality-of-Life Questionnaire-Emotional Function; MSQ-P: Migraine-Specific Quality-of-Life Questionnaire-Role Function-Preventive; MSQ-R: Migraine-Specific Quality-of-Life Questionnaire-Role Function-Restrictive; SD: standard deviation.*

### 3.2 Degree centrality difference between migraine patients and healthy controls

By using spatial distribution maps, the functional hubs were highly similar in the two groups (high DC), which localized in the Limbic Lobe and Frontal Lobe (Fig. 1), and intergroup differences (Fig. 1, see **Supplementary Tables 1,2,3**) were also remarkably similar based on the different correlation thresholds (r_0_ = 0.15, 0.2, 0.25 and 0.3). Therefore, we mainly reported the results for DC when the correlation threshold was 0.25 in a bina graph. Compared to healthy controls, migraine patients exhibited an increased DC in the medial superior frontal gyrus (SFG) of right hemisphere and anterior cingulate cortex (ACC) of both hemispheres. Among these clusers, migraine patients exhibited a significantly increased DC in one clusters and this cluster was located in the left ACC (Table 3, Figs. 2,3). These changes of the DC overlapped with the functional hubs.

We tried to find out the association between values of DC and clinical variables, examined by Pearson’s correlation analysis with a statistical significance level of *p *< 0.05. However, no significant correlation was found between the increased DC and the clinical variables.

**Fig. 1.** Compared to healthy controls, migraine patients showed remarkably similar altered DC brain areas according to different correlation thresholds (r_0_ = 0.15, 0.2, 0.25 and 0.3) (the permutation test with FDR corrected *p *< 0.05). The hot (cool) color indicates significantly increased(decreased) DC brain area. Abbreviations: DC: degree centrality; L (R): left (right) hemisphere.

**Table 3.** Significant differences in degree centrality between the patients with migraine patients and healthy controls (r_0_ = 0.25).

Condition	L/R	Brain regions	MNI coordinates			Intensity	Cluster size (Voxle)
			X	Y	Z		
MP > HC	L	Anterior Cingulate Cortex	−6	33	27	4.4596	113
MP > HC	R	Anterior Cingulate Cortex	9	30	30	3.9701	4
MP > HC	R	Superior frontal gyrus, medial	3	21	45	4.1661	6

*Abbreviations: MP: migraine patients; HC: healthy controls; L (R): left (right) cerebral hemisphere; MNI: Montreal Neurological Institute.*

**Fig. 2.** Compared to healthy controls, migraine patients showed that increased DC brain areas when r_0_ was 0.25. The hot (cool) color indicates significantly increased (decreased) DC brain area. The hot (cool) color indicates significantly increased (decreased) DC brain area. Abbreviations: ACC-L(R): anterior cingulate cortex of left(right) hemisphere; SFG-R: superior frontal gyrus of right hemisphere.

**Fig. 3.** Bar plot of DC for the significant increased clusters between migraine patients and healthy controls in the left ACC (A), the right ACC (B) and the right SFG (C). Abbreviations: Patients: migraine patients; HC: healthy controls; SD: standard deviation; DC: degree centrality; ACC-L(R): anterior cingulate cortex of left(right) hemisphere; SFG-R: superior frontal gyrus of right hemisphere. *: each bar chart and line segment perpendicular to it represent the mean and standard deviation, respectively.

### 3.3 FC differences between groups and FC-related correlation analyses

According to the above results of DC, we performed seed-based FC analysis based on the left ACC. Compared with healthy controls, migraine patients showed increased FC between the left ACC and the right SFG and decreased FC between the left ACC and the left superior temporal gyrus (STG) (Table 4 and Fig. 4). The effects are significant at a single voxel *p *< 0.01, FDR corrected *p *< 0.05. As shown in Fig. 5, the Pearson’s correlation analysis demonstrated that the FC value of the right SFG was positively correlated with the score of migraine-specific quality-of-life questionnaire about role in function-preventive (MSQ-P) (*r* = 0.447, *p* = 0.01) in migraine patients.

**Table 4.** FC comparisons between migraine patients and healthy controls.

Condition	L/R	Brain regions	MNI coordinates			Intensity	Cluster size (Voxel)
			X	Y	Z		
MP < HC	L	Superior Temporal Gyrus	−54	−21	−3	−5.1207	9
MP > HC	R	Superior Frontal Gyrus	21	9	57	5.0113	333

*Abbreviations: MP: migraine patients; HC: healthy controls; L (R): left (right) cerebral hemisphere; MNI: Montreal Neurological Institute.*

**Fig. 4.** FC results of migraine patients and HCs (FDR corrected). Compared with HCs, migraine patients showed decreased FC between the left ACC and the right STG (A, B) and increased FC between the left ACC and the right SFG (A, C). Scatter plot of FC values for the significantly altered regions between migraine patients and healthy controls (*p *< 0.05). The hot (cool) color indicates significantly increased (decreased) DC brain area. Abbreviations: FC: functional connectivity; patients, migraine patients; HCs: healthy controls; ACC-L: anterior cingulate cortex of left hemisphere; STG-L: superior temporal gyrus of left hemisphere; SFG-R: superior frontal gyrus of right hemisphere; SD: standard deviation; *: each bar chart and line segment perpendicular to it represent the mean and standard deviation, respectively.

**Fig. 5.** The correlations between the score of MSQ-P and FC in the SFG-R. Abbreviations: FC: functional connectivity; SFG-R: superior frontal gyrus of right hemisphere; MSQ-P: Migraine-Specific Quality-of-Life Questionnaire-Role Function-Preventive; SD: standard deviation.

## 4. Discussion

In our study, we investigated the intrinsic functional hubs changes across the whole brain using voxel-wise DC in migraine patients and found that DC was significantly increased in the left ACC, slightly increased in the right ACC and right SFG, suggesting that these areas have high function connection. These changes may be a secondary and adaptive change due to the relevant brain damages in the migraine patients. In addition, the seed-based FC analysis based on the left ACC showed that the strength of the functional connection in migraine patients was higher in the right SFG but lower in the left STG. The strengthened functional connection of the right SFG was positively correlated with the score of MSQ-P.

Recently, the topological organization of brain networks is gradually regarded as the physiological basis of mental representation and information processing [39]. As an increasingly useful tool, graph theoretical analysis has been used to describe the complex system of functional brain networks, and provides a unique framework for identifying differences between the topological organization of brain networks [40]. Accordingly, many studies have used this kind of tool and theory in their method. For example, a comparative study about PAG resting state FC made clever use of graph theoretical analysis and succeeded to find that migraine patients showed decreased resting state FC between the PAG and rostral ACC/medial prefrontal cortex, which are crucial regions in the descending pain modulatory system [41].

Currently, the voxel-wise degree centrality (DC) plays an important role in superior propagation, information integration and critical way stations for processing, which represents the overall connectivity between particular brain voxels to other brain voxels [42, 43, 44]. Voxel-wise DC is a network measurement tool at the voxel level based on graph theory, and DC value is an important metrics describing the number of direct connections for a given voxel with the rest of the whole brain voxel rather than between specific nodes or regions. The brain functional hubs are brain regions concentrated by large number of connections with the rest of whole brain. Functional hubs usually exhibit higher metabolic demands and longer-distance connections [45] and are especially more vulnerable to aberrant disease conditions than non-hubs [46]. Therefore, the application of voxel-wise DC can better represent the migraine-related brain functional hubs without a priori nodes or a region of interest [30]. Previous research has also confirmed that voxel-wise DC has a high sensitivity, specificity, and test-retest reliability [47], and it has been widely use in neurodegenerative disorders and psychiatric [47, 48, 49, 50, 51, 52]. In study design and mothed, we used the comparison of functional connectivity between healthy people and migraine patients to explore the spatial centrality of the whole brain functional network related to migraine and to investigate the potential functional hubs associated with migraine. In the other hand, we adopted the permutation test (5000 times) with False Discovery Rate (FDR) for multiple comparisons, which is consider as relatively strict correction and conducive to the analysis of results.

As is known to all, ACC is a crucial brain structure that not only participates in analgesia mechanisms, but also participates in the emotional dimensions of pain, such as self-related negative emotional states [53]. Likewise, ACC is largely involved in numerous processes in migraine behaviors [54], such as executive function [55], interoception [56], pain modulation [57], salience [58], amongst others. The results of our study also revealed that ACC is one of the main cortical hubs in the brain network affected by migraine. To some extent, this finding is consistent with the previous studies that showed the involvement of the ACC in migraine. Two previous studies [59, 60] about resting-state functional MRI showed that migraine patients had significant functional connectivity reduction in the dorsal anterior cingulate cortex. Structurally, Yang Yu *et al*. [61] performed voxel-based morphometry analysis to assess gray matter volume differences among groups and found migraine groups showed decreased volume of gray matter in the ACC compared with the HC group. In another study, Ting Xue *et al*. [62] found that migraine patients presented decreased amplitude of low-frequency fluctuation (ALFF) values in the anterior cingulate cortex compared with healthy persons. In the current study, we observed diminished DC value in migraine patients no matter in the left or right ACC. Therefore, our finding might speculate that the ACC is a sensitive region in the pathological process of migraine.

Anxiety and depression were more closely associated with increase in migraine risk than other factors [63, 64, 65]. Lack of ability to properly control anxiety and relax oneself is the most prominent problem in migraine psychiatric comorbidities [66]. Physical symptoms in depression show more association with migraine than emotional symptoms [67]. In our study, migraine patients showed high scores of SAS and SDS than healthy controls, which means that migraine patients are more likely be anxious and depressed. Thus, we speculate that as a kind of negative emotional stimuli, anxiety and depression largely results in the increase of DC value in the ACC. Interestingly, we found that there were obvious differences in DC value between left and right ACC and this may have something to do with a temperamental disposition to fear and anticipatory worry [68]. However, we found that DC value had no correlation with clinical data, which will be supplemented by large sample studies in the future.

Same as previous studies [13, 14], we also noticed the change in the ACC and as a complement, we found that migraine patients possessed increased FC between the left ACC and the right SFG and decreased FC between the left ACC and the left STG. Meanwhile, FC in the right SFG also presented positive correlation with the score of MSQ-P. We infer that different sample sizes and imaging modalities may be responsible for the discrepancy.

Previous studies have confirmed that the medial SFG is play a key role in participative feelings of pain and emotional responses, including attention response, cognitive reaction and memory related to pain [69, 70]. The prefrontal cortex (PFC) consisting of the superior frontal gyrus, the middle frontal gyrus and the inferior frontal gyrus refers to the entire frontal cortex except the primary and the secondary motor cortex, which is the regulatory region associated with pain-relief of opioids and other forms of analgesia and can reduce the perception of pain signals under the influence cognitive regulation [71, 72, 73, 74]. A study [75] about pain-induced stimuli showed that migraine can generate more activation in the PFC and other brain regions associated with pain. Therefore, the enhancement of emotional responses and participative feelings of pain, such as pain-related anxiety and depress may affect brain sensitivity, especially SFG and this kind of high alertness of pain may be the reason why migraine can cause a change in DC value. Besides, both ACC and SFG are in charge of emotional process together, there is an increased functional connection between the two gyrus, ultimately having an impact on the migraine patients’ performance of normal activities. To supplement this, we found that increased FC between the left ACC and the right SFG might reflect a relationship with migraine.

In our migraine patients, the left STG showed decreased FC with the left ACC. Many previous studies [76, 77, 78] had found that volume of gray matter was increased in bilateral STG of patients with tinnitus and confirmed that the tinnitus would cause corresponding changes in the STG. In a perfusion functional MRI study about tinnitus patients with migraine, Zhengui Xu *et al*. [79] observed not only that the tinnitus patients exhibited decreased relative cerebral blood flow (rCBF) in the right STG, but also that the rCBF in the STG of migraine patient with tinnitus would be obviously decreased. Therefore, we speculate that decreased FC between the STG and ACC may have something to do with migraine-related aura phenomenon, especially auditory aura.

In our study, some limiting factors should be considered. Firstly, we recruited 55 healthy controls and 32 migraine patients, which is a relatively small sample and our study was a single-center study and the results may not be representative of functional connectivity in migraine patients of all regions. Based on these, the results of our study need to be further verified in large-sample multicenter studies. Secondly, our study was a cross-sectional study. We only analyzed brain functional connectivity of migraine patients at one point in time, but did not follow up migraine patients. Thus, we cannot analyze the functional connectivity changes throughout the course of migraine and the changes in DC value of ACC as the disease progresses or is treated. Thirdly, the gender ratio in patient group is imbalanced and we will pay attention to the recruitment of male migraine patients and avoid imbalanced gender in future studies. Finally, DC is the number of direct connections for a node and edges, which represents the local quantifiable measure of a metrics index without an eigenvector. So the qualitative change of global information need to be further explored by using voxel-wise eigenvector centrality [30].

## 5. Conclusions

In conclusion, this is a study aiming to investigate the intrinsic functional hubs changes between migraine patients and healthy people by using voxel-wise DC. The ACC and right SFG have higher function connection with increased DC value. Further, the seed-based FC analysis suggests that the left ACC in migraine patients has prominent functional connection with the right SFG but lower with the left STG.

## 6. Key findings & clinical implications

Based on the analysis of relatively changed DC, our study has identified the presence of abnormal intrinsic functional hubs in the brains of migraine patients. These findings hold promising implications for developing future treatment strategies and refining patient care for migraine. These findings also expand our understanding of the functional characteristics of migraine, and may provide an insight into understanding the dysfunction and pathophysiology of migraine patients. By identifying these specific abnormalities, healthcare professionals can tailor treatment plans to target these key neural hubs, potentially leading to more effective and personalized interventions. Additionally, these insights provide valuable strategical considerations for addressing the complexities of migraine management.

## Supplementary Material


