# Supplementary material for: Abnormal functional hubs in migraine patients: a resting-state MRI analysis about voxel-wise degree centrality
Source: J Oral Facial Pain Headache. 2024 Mar 12;38(1):52–63. doi: 10.22514/jofph.2024.006 (PMC11774280; doi:10.22514/jofph.2024.006)
Supplement: Supplementary file 1 [file Supplementary-material.docx]

Supplementary material

Supplementary Table 1. Significant differences in Degree Centrality between migraine patients and healthy controls (r_0_ = 0.15).

| Condition | L/R | Brain regions | MNI coordinates | | | Intensity | Cluster size  (Voxle) |
| --- | --- | --- | --- | --- | --- | --- | --- |
|  |  |  | X | Y | Z |  |  |
| MP < HC | L | Fusiform_L (aal) | −18 | −69 | −18 | −4.2208 | 13 |
| MP > HC | R | Inferior Frontal Gyrus | 30 | 27 | −3 | 5.2974 | 40 |
| MP > HC | R | Caudate_R (aal) | 12 | 15 | 0 | 4.8843 | 60 |
| MP > HC | L | Thalamus | −9 | −3 | 6 | 4.1032 | 8 |
| MP > HC | L | Caudate_L (aal) | −15 | 18 | 3 | 3.6371 | 2 |
| MP > HC | L | Caudate_L | −12 | 9 | 6 | 3.7804 | 4 |
| MP > HC | L | Cingulum_Ant_L | 0 | 42 | 18 | 4.6259 | 383 |
| MP > HC | L | Frontal_Mid_L | −27 | 24 | 45 | 4.9649 | 17 |

MP: migraine patients; HC: healthy controls; L (R): left (right) cerebral hemisphere.

Supplementary Table 2. Significant differences in Degree Centrality between migraine patients and healthy controls (r_0_ = 0.20).

| Condition | L/R | Brain regions | MNI coordinates | | | Intensity | Cluster size  (Voxle) |
| --- | --- | --- | --- | --- | --- | --- | --- |
|  |  |  | X | Y | Z |  |  |
| MP > HC | R | Caudate_R (aal) | 12 | 15 | 0 | 4.6964 | 19 |
| MP > HC | R | Inferior Frontal Gyrus | 30 | 27 | −3 | 4.9522 | 18 |
| MP > HC | L | Anterior Cingulate | −6 | 36 | 27 | 4.5885 | 291 |

MP: migraine patients; HC: healthy controls; L (R): left (right) cerebral hemisphere.

Supplementary Table 3. Significant differences in Degree Centrality between migraine patients and healthy controls (r_0_ = 0.30).

| Condition | L/R | Brain regions | MNI coordinates | | | Intensity | Cluster size  (Voxle) |
| --- | --- | --- | --- | --- | --- | --- | --- |
|  |  |  | X | Y | Z |  |  |
| MP > HC | L | Anterior Cingulate | −3 | 27 | 18 | 4.4404 | 1 |
| MP > HC | L | Anterior Cingulate | −6 | 33 | 27 | 4.3021 | 4 |

Abbreviations: MP: migraine patients; HC: healthy controls; L (R): left (right) cerebral hemisphere.
